# COVID-19 Associated with Cryptococcosis: A New Challenge during the Pandemic

**DOI:** 10.3390/jof8101111

**Published:** 2022-10-21

**Authors:** Khee-Siang Chan, Chih-Cheng Lai, Wen-Liang Yu, Chien-Ming Chao

**Affiliations:** 1 Department of Intensive Care Medicine, Chi Mei Medical Center, Yongkang, Tainan 71004, Taiwan; 2 Division of Hospital Medicine, Department of Internal Medicine, Chi Mei Medical Center, Tainan 71004, Taiwan; 3 School of Medicine, College of Medicine, National Sun Yat-sen University, Kaohsiung 80424, Taiwan; 4 Department of Medicine, School of Medicine, College of Medicine, Taipei Medical University, Taipei 11031, Taiwan; 5 Department of Intensive Care Medicine, Chi Mei Medical Center, Liouying, Tainan 73657, Taiwan; 6 Department of Dental Laboratory Technology, Min-Hwei College of Health Care Management, Tainan 73657, Taiwan

**Keywords:** COVID-19, co-infection, cryptococcosis, mortality, SARS-CoV-2

## Abstract

Coronavirus disease 2019 (COVID-19) caused by severe acute respiratory syndrome coronavirus 2 (SARS-CoV-2) is a great threat to global health. In addition to SARS-CoV-2 itself, clinicians should be alert to the possible occurrence of co-infection or secondary infection among patients with COVID-19. The possible co-pathogens include bacteria, viruses, and fungi, but COVID-19-associated cryptococcosis is rarely reported. This review provided updated and comprehensive information about this rare clinical entity of COVID-19-associated cryptococcosis. Through an updated literature search till 23 August 2022, we identified a total of 18 culture-confirmed case reports with detailed information. Half (n = 9) of them were elderly. Fifteen (83.3%) of them had severe COVID-19 and ever received systemic corticosteroid. Disseminated infection with cryptococcemia was the most common type of cryptococcosis, followed by pulmonary and meningitis. Except one case of *C. laurentii*, all other cases are by *C. neoformans*. Liposomal amphotericin B and fluconazole were the most commonly used antifungal agents. The overall mortality was 61.1% (11/18) and four of them did not receive antifungal agents before death. Improving the poor outcome requires a physician’s high suspicion, early diagnosis, and prompt treatment.

## 1. Introduction

Since early 2020, coronavirus disease 2019 (COVID-19) rapidly posed a great threat to public health and has been declared a pandemic by World Health Organization (WHO) [1,2]. As of 18 October 2022, there have been 622,389,418 confirmed cases of COVID-19, including 6,548,492 deaths, reported to WHO [3]. COVID-19 is caused by severe acute respiratory syndrome coronavirus 2 (SARS-CoV-2), and its clinical presentations can range from asymptomatic, mild disease to severe or acute respiratory distress syndrome [4]. However, in addition to SARS-CoV-2, clinicians should be alert to the possible occurrence of co-infection or secondary infection among patients with COVID-19, especially for critical diseases [5,6,7].

Both bacteria, including *Streptococcus pneumoniae*, *Staphylococcus aureus*, *Klebsiella pneumoniae*, *Mycoplasma pneumoniae*, *Chlamydia pneumonia*, *Legionella pneumophila*, and viruses including influenza, coronavirus, rhinovirus/enterovirus, parainfluenza, metapneumovirus, and human immunodeficiency virus (HIV) have been reported as co-pathogens. However, the COVID-19-associated fungal infections caused by *Candida*, *Aspergillus*, and *Mucorales* cannot be neglected [5,8,9,10,11,12,13,14,15]. Recently, several cases of COVID-19 with *Cryptococcus* co- or secondary infection as opportunistic infections have been reported [16,17,18,19,20,21,22,23,24,25,26,27,28,29,30,31,32,33,34,35], However, many issues about the epidemiology, pathogenesis, clinical and microbiological manifestations, and treatment of COVID-19-associated cryptococcosis remained unclear due to limited studies. Therefore, we did a literature search using the keywords—“cryptococcus,” “opportunistic infection,” “COVID-19,” and “SARS-CoV-2,” and conducted this review to provide updated and comprehensive information about this rare COVID-19-associated cryptococcosis.

## 2. Epidemiology

To determine the incidence of and examine factors associated with cryptococcosis after a diagnosis of COVID-19, Chastain and his colleagues used TriNetX—a global federated research network that captures anonymous data from electronic medical records of 57 healthcare organizations and included a total of 212,479 hospitalized patients with COVID-19 [18]. Based on ICD-10-CM diagnosis codes, they identified 65 patients with cryptococcosis, and the overall incidence and prevalence of cryptococcosis among hospitalized patients with COVID-19 were 0.022% and 0.059%, respectively [18]. Compared to non-Hispanic patients, the incidence and prevalence were higher among Hispanic or Latino populations (incidence: 0.029% vs. 0.022%; prevalence: 0.063% vs. 0.061). In addition, male patients had a higher incidence (0.029%) and prevalence (0.063%) of cryptococcosis after COVID-19 than female patients [18]. Finally, a higher prevalence of cryptococcosis was observed in two specific groups, including the elderly group (0.043% among patients 60–64 years and 0.035% among those 70–74 years) and patients with critical COVID-19 requiring ICU admission (0.123%) [18]. Another small series of 13 patients with confirmed cryptococcal infection following COVID-19 reported a similar finding that 12 (92.3%) patients had severe COVID-19 [16].

Compared to COVID-19 patients without cryptococcosis (n = 212,414), hospitalized patients with COVID-19-associated cryptococcosis (n = 65) were more likely to be male (80% vs. 51%) and had more underlying comorbidities such as HIV infection (32% vs. 2%), transplanted organ (28% vs. 3%), immunodeficiency with predominantly antibody defect (15% vs. <1%), other immunodeficiencies (32% vs. 3%), sarcoidosis (15% vs. <1%), systemic connective tissue disorder (15% vs. 4%), rheumatoid arthritis (15% vs. <1%), non-infective enteritis and colitis (15% vs. 5%), hepatic fibrosis and cirrhosis (15% vs. 3%), type 2 diabetes mellitus (42% vs. 30%), heart failure (28% vs. 17%), chronic kidney disease (40% vs. 24%), and malnutrition (26% vs. 5%) (all *p* < 0.05) [18]. Many reported cases had underlying diseases, such as obesity, diabetes mellitus, hypertension, ischemic heart disease, HIV infection, organ transplant recipient, liver cirrhosis, chronic obstructive pulmonary disease, chronic kidney disease, and autoimmune hemolytic anemia [18,19,20,21,24,25,26,27,28,29,30,31,32,33,34]. Through an updated literature search till 23 August 2022, we identified a total of 18 culture-confirmed case reports with detailed information (Table 1). We had the similar findings that half (n = 8) of them were elderly and had underlying diseases, particularly for diabetes mellitus (n = 7). However, some patients did not have underlying diseases [17,23].

Anti-inflammatory agents, such as corticosteroid, interleukin-6 blockade, and Janus kinase (JAK) inhibitors, are commonly used and recommended for hospitalized patients with severe COVID-19 [36,37,38,39]; however, these agents may be associated with the risk of infections. One small case series reported that 12 patients (92.3%) and three patients (23%) among 13 patients who developed a cryptococcal infection after COVID-19 had ever received corticosteroids equivalent to prednisone 10 mg/day for more than 7 days and tocilizumab, respectively [16]. Compared to patients without cryptococcosis among hospitalized patients with COVID-19, those with cryptococcosis were significantly more likely to have received tocilizumab (odds ration [OR], 18.6; 95% confidence interval [CI], 9.5–36.3, *p* < 0.0001) or baricitinib (OR, 12.4; 95% CI, 6.4–24.1, *p* < 0.0001), but not dexamethasone (OR, 0.7; 95% CI 0.4–1.2, *p* = 0.222) [18]. In addition, the uses of immunosuppressants before COVID-19 such as tacrolimus, cyclophosphamide, and prednisone have been observed in previous case reports [25,30,34]. Similarly, fifteen (83.3%) of them had severe COVID-19 and ever received systemic corticosteroid in our review of 18 cases (Table 1).

## 3. Pathogenesis

Immune dysregulation and cumulative risk factors about immune, cellular, and virological presentation may lead to fatal outcomes caused by secondary bloodstream infections of opportunistic *Cryptococcus* spp. [25]. The pathogenic *Cryptococcus* enters the human host via inhalation into the lung, where the environment is limiting in serum opsonin and the mechanism of nonopsonic phagocytosis occurs via phagocytes in such situations [40]. In addition, the cell walls and capsules of *Cryptococcus* are rich in immunomodulatory polysaccharides of glucuronoxylomannan, which is shed from the fungus and circulates in the blood and cerebrospinal fluid of patients with cryptococcosis [41]. The uptake of nonopsonized *Cryptococcus neoformans* (via both dectin-1 and dectin-2) and *Cryptococcus gattii* (largely via dectin-1) through the mannose receptor is dependent on macrophage activation by cytokines. Dectin-2, a C-type lectin receptor that recognizes high-mannose polysaccharides, is involved in cytokine production, actin polymerization of cytoskeletons, engulfment of *C. neoformans*, and phagocytosis by the bone marrow-derived dendritic cell in response to stimulation with *C. neoformans* [42].

Cryptococcal mannoproteins are capable to bind to the conserved mannose receptor, CD206, on dendritic cells [43], and the dendritic cell is the most efficient antigen-presenting cell for *C. neoformans* mitogen in presenting to T cells. Therefore, efficient T cell responses to *C. neoformans* mannoproteins require recognition of terminal mannose groups by human macrophage mannose receptors. Individuals with compromised T cell function are susceptible to cryptococcosis [44,45,46]. In addition, the fungal pathogen *C. neoformans* invades the brain through a “Trojan Horse” mechanism, whereby the fungus crosses the blood–brain barrier as a transcellular passenger inside host phagocytes [47,48].

Finally, although the precise interaction between SARS-CoV-2 and *Cryptococcus* remains unknown, the most likely pathophysiology is multifactorial involving cytokine dysregulation and impaired cell-mediated immunity due to SARS-CoV-2 and the use of immunomodulatory therapies in the treatment of COVID-19. In addition to the role of corticosteroids in COVID-19-associated cryptococcosis, several studies found that SARS-CoV-2 infection may affect T-cell response in multiple ways including lymphopenia, and the reduction in T cells, especially in severe COVID-19 [49,50,51,52]. Specifically, both CD4+ and CD8+ T cells were lower in patients with SARS-CoV-2 infection [49,50,53]. This relative deficiency in cell-mediated immunity may increase the susceptibility to cryptococcal infection.

## 4. Clinical Manifestations

According to a multicenter research network study by Chastain et al., most episodes of cryptococcosis developed within 10 days after COVID-19 diagnosis [18]. Similarly, Regalla et al. reported that in addition to two patients who had cryptococcosis diagnosed postmortem, the median time duration between COVID-19 diagnosis and *Cryptococcus* infection was 13 days (interquartile range [IQR], 5–49) in 11 patients [16]. Choi reported rare unusual presentation in an immunocompetent patient who developed culture-confirmed pulmonary cryptococcosis three months after COVID-19 [17]. Pulmonary and cerebral cryptococcosis were the most common form of COVID-19-associated cryptococcal infections, followed by disseminated and cutaneous infections [16,18]. Deepa et al. reported rare cryptococcal endophthalmitis [19].

Based on our analysis of 18 cases with detail data, disseminated infection, defined by either a positive blood culture or the involvement of cryptococcosis at least two different sites, was the most common type of cryptococcosis, followed by pulmonary and meningoencephalitis (Table 1). For patients with pulmonary cryptococcosis, the clinical manifestations include fever, cough, shortness of breath, hypotension, respiratory insufficiency, and hypoxia [16,17,28]. For patients with cryptococcal meningoencephalitis, patients may present with fever, headache, fatigue, vomiting, presyncope, unsteady gait, frequent falls, aphasia, agitation, somnolence, obtundation, impaired memory, delayed verbal responses, inattention, and confusion [16,23,27,31,34]. Patients with disseminated *Cryptococcus* infection, including cryptococcemia, may present with lethargy, hypoxia, septic shock, dyspnea, and multiorgan dysfunction [20,24,25,32,33].

However, because both COVID-19 and cryptococcosis can present with these nonspecific symptoms, it is difficult to diagnose cryptococcosis among patients with COVID-19 based on the clinical information only. Based on the present evidence, the best way to diagnose cryptococcosis in patients with COVID-19 should require the clinician’s vigilance and further microbiological investigation.

## 5. Microbiologic Investigation

Among patients with COVID-19-associated cryptococcosis, *C. neoformans* was the most common reported causative pathogen in the culture-confirmed cases [17,20,21,22,23,25,26,27,32], and one case of endophthalmitis was caused by *Cryptococcus laurentii* [19]. Additionally, co- or secondary infections with other microorganisms have been reported in patients with COVID-19-associated cryptococcosis, and the reported pathogens included methicillin-resistant *Staphylococcus aureus* (MRSA) [26], *Leclercia adecarboxylata* [22], multidrug-resistant *Enterobacter cloacae* [21], *Alcaligenes* spp. [21], *Candida parapsilosis* [24], *Klebsiella* spp. [19], and *Aspergillus fumigatus* [30]. Among these co-pathogens, *L. adecarboxylata* [22], multidrug-resistant *E. cloacae* [21], and *Alcaligenes* spp. [21]. developed after cryptococcosis, but MRSA [26], *C. parapsilosis* [24], and *Klebsiella* spp. [19], developed before cryptococcosis. Only *A. fumigatus* had co-infection with cryptococcus at the same time [30].

## 6. Laboratory and Radiologic Findings

Although there was no significant difference in terms of the leukocyte and lymphocyte counts between COVID-19 patients with and without cryptococcosis, patients with cryptococcosis had significantly lower CD4 cell counts than those without cryptococcosis (73.9 ± 68.9 cells/μL vs. 299 ± 316 cells/μL, *p* = 0.0242) [18]. In addition, patients with cryptococcosis had significantly higher alanine aminotransferase, alkaline phosphatase, ferritin, and lactate dehydrogenase, but lower albumin than those without cryptococcus (all *p* < 0.05) [18].

The radiographic findings among patients with pulmonary cryptococcosis included nodule, subpleural cavitated lesion, ground glass opacity, and consolidation. For patients with cerebral cryptococcosis, brain images may show hydrocephalus, abnormal hyperintense foci in the basal ganglia, and numerous acute and subacute infarcts in the cerebral and cerebellar hemisphere; however, normal brain images have been reported in patients with cryptococcal meningoencephalitis.

Although these laboratory and radiological findings, such as lower CD4 counts, and brain abnormalities may hint the possible cryptococcosis in patients with COVID-19, some radiological findings, such as hydrocephalus could be delayed findings.

## 7. Diagnosis

Cryptococcosis should be considered in patients with severe COVID-19 infection who develop new clinical deterioration or worsening respiratory status, especially in immunocompromised patients or severe COVID-19 patients who received high-dose steroids and tocilizumab [16,17,29,31]. There are challenges regarding the diagnosis of COVID-19-associated opportunistic cryptococcosis due to the limited experience about this rare clinical entity [16]. Overall, diagnosis of cryptococcosis following COVID-19 requires clinical alertness and can be confirmed by culture, antigen test, and histopathological findings. In our review of 18 cases (Table 1), all the cases were confirmed by culture and seven of them have tested positive for cryptococcus antigen. Among these tests, the serum cryptococcal antigen test can help facilitate the accurate and rapid diagnosis of cryptococcosis [54]. Since three reported cases were diagnosed as cryptococcal infection postmortem [20,25,26], high suspicion and aggressive diagnostic testing should be essential for early diagnosis and appropriate treatment. Because of the immunologic dysfunction associated with SARS-CoV-2 infection, all patients with cryptococcemia undergo evaluation for dissemination to the central nervous system.

Pulmonary cryptococcosis should be considered in the differential diagnosis of solitary or multiple pulmonary nodules in patients with COVID-19 [17]. While air bronchograms within nodules or masses with a predominant distribution in the lower lung lobes and peripheral area are more frequently present in immunocompetent patients [55,56,57]. Cavitations within nodules or masses are more frequently present in immunocompromised patients than in immunocompetent patients [55,56].

## 8. Treatment

Among the recently reported case of COVID-19-associated cryptococcosis, liposomal amphotericin B with or without flucytosine followed by fluconazole were the most commonly used antifungal treatment (Table 1) [23,27,29,31,32]. This regimen is consistent with the recommendation of the Infectious Diseases Society of America [58,59]. For immunocompetent COVID-19 patients with CNS involvement, standard therapy consists of combination therapy with amphotericin B (0.7–1 mg/kg/d) plus flucytosine (100 mg/kg/d) for at least 4–week induction therapy, followed by fluconazole (400 mg/day) for a 8–week consolidation therapy, and then fluconazole (200 mg/day) as maintenance therapy for 6–12 months [59]. Alternative induction therapy may include amphotericin B (0.7–1 mg/kg/d) for 6–10 weeks for flucytosine–intolerant patients, or lipid formulation of amphotericin B (3–5 mg/kg/d) for 6–10 weeks for patients with significant renal disease [59]. A lumbar puncture is recommended after 2 weeks of treatment to assess the status of cerebrospinal fluid sterilization. Patients with a positive culture at 2 weeks may require a longer course of induction therapy.

For those healthy individuals with non-CNS-isolated cryptococcemia or evidence of high fungal burden based on positive serum cryptococcal antigen titer ≥ 1:512, the recommended treatment is to treat the disease like CNS involvement [59]. For immunocompetent hosts with the isolated pulmonary disease with mild-to-moderate symptoms, indicated treatment is fluconazole, 200–400 mg/day for 6 months, which may be shortened to 3 months or extended to 12 months, based on the resolution of the disease. For those individuals who are unable to tolerate fluconazole, itraconazole (200–400 mg/day for 6 months) or amphotericin B, 0.5–1 mg/kg/d (total, 1000–2000 mg), is an acceptable alternative. For patients with more severe diseases, treatment may be necessary like CNS disease [59]. Overall, treatment of cryptococcal infection among patients with COVID-19 is prescribed on the basis of the severity of the disease and the degree of immunosuppression (Table 2).

## 9. Outcome

The outcome of COVID-19-associated cryptococcosis was poor [16,18,35]. One series involving 13 cases showed that 12 patients (92.3%) required mechanical ventilation, and all patients (n = 13, 100%) required ICU admission [16]. The overall mortality was 53.8% (7/13) [16]. Similarly, our review of 18 cases showed that the high mortality of 61.1% (n = 11) and four of them did not receive antifungal agents before death (Table 1). To improve the clinical outcomes of COVID-19-associated cryptococcosis, clinicians need to be vigilant about the possible occurrence of cryptococcal infection among patients with SARS-CoV-2 infection and arrange for appropriate tests to early diagnosis and prompt treatment.

## 10. COVID-19 Associated with Cryptococcosis among HIV Patients

In our review of 18 cases, only one case of COVID-19-associated cryptococcosis developed in patients with HIV [27], who present with *C. neoformans* meningoencephalitis and had survival discharge after treatment of liposomal amphotericin-B, followed by fluconazole (Table 1). In the series of 65 cases with COVID-19-associated cryptococcosis, 21 (32%) patients had underlying HIV infection [18]. Among them, cerebral cryptococcosis was the most common reported form (n = 20, 63%). About their outcomes, Chastain et al. reported that the patients developed cryptococcosis had significantly higher risk of mechanical ventilation requirement (42% vs. 6%, *p* < 0.0001), ICU admission (50% vs. 19%, *p* < 0.0001), and mortality (42% vs. 18%, *p* = 0.0030) than those without cryptococcosis [18].

## 11. Conclusions

In summary, COVID-19-associated cryptococcosis is uncommon; however, it could be underestimated due to underdiagnosis. The clinical manifestations are non-specific and diagnosis requires laboratory examinations. Clinicians should keep in mind this differential diagnosis, especially for patients with severe COVID-19 who received corticosteroids. Improving the poor outcome requires a physician’s high suspicion, early diagnosis, and prompt treatment.

## Figures and Tables

**Table 1:** Clinical characteristics of the 18 reported cases with COVID-19-associated cryptococcosis.

Case (Country)	Age	Sex	Underlying Disease	COVID-19 Severity	Use of Corticosteroid for COVID-19	Cryptococcal Infection					
						Sites of Involvement	Diagnosis	Pathogen	Treatment	Outcome	Timing of Diagnosis, Day
Abohelwa et al. [28] (USA)	78	F	HTN, DM	Severe	NA	Pulmonary	Tracheal aspirate Cx	*C. neoformans*	FLZ	Dead	NA
Alegre-Gonz’alez et al. [29] (Spain)	78	M	HTN, DM, CKD	Severe	Yes	Disseminated	Blood Cx, CSF Cx, CSF CrAg	*C. neoformans*	L-AmB + FC -> FLZ	Dead	D75
Cafardi et al. [21] (USA)	78	M	HTN, COPD	Severe	Yes	Pulmonary	BAL Cx,	*C. neoformans*	L-AmB -> ISZ	Dead	D22
Chastain et al. [20] (USA)	70+	M	HTN, COPD, CKD, CAD, stroke, obesity	Severe	Yes	Disseminated	Blood Cx	*C. neoformans*	Nil	Dead	Postmortem
Choi et al. [17] (Korea)	46	M	Nil	Mild	NA	Pulmonary	BAL Cx and Cr Ag	*C. neoformans*	FLZ	Alive	D90
Deepa et al. [19] (India)	50+	M	DM	NA	Yes	Ocular	Vitreous Cx	*C. laurentii*	FLZ + Intravitreal VCZ	Alive	NA
Gamon et al. [22] (German)	55	M	Dilated cardiomyopathy	Severe	Yes	Pulmonary	Respiratory specimen Cx	*C. neoformans*	L-AmB -> FLZ	Alive	D13
Ghanem et al. [23] (USA)	73	F	None	Severe	Yes	Meningeal	CSF Cx and CrAg	*C. neoformans*	L-AmB + FC -> FLZ	Alive	D7
Gil et al. [33] (USA)	59	M	HTN, DM, obesity	Severe	Yes	Disseminated	Blood Cx	*C. neoformans*	L-AmB -> FLZ	Alive	D33
Heller et al. [27] (USA)	24	M	HIV	Mild	No	Meningeal	CSF Cx and CrAg	*C. neoformans*	L-AmB + FC -> FLZ	Alive	D5
Karnik et al. [32] (USA)	57	M	HTN	Severe	Yes	Disseminated	Blood Cx, CSF Cx and CrAg	*C. neoformans*	L-AmB + FC	Dead	D36
Khatib et al. [24] (Qatar)	60	M	HTN, DM, ischemia heart disease	Severe	Yes	Disseminated	Blood Cx	*C. neoformans*	L-AmB + FC	Dead	D48
Passarelli et al. [25] (Brazil)	75	M	HTN, renal transplant	Severe	Yes	Disseminated	Blood Cx	*C. neoformans*	Nil	Dead	Postmortem
Thota et al. [31] (USA)	76	F	HTN	Severe	Yes	Disseminated	Blood Cx, CSF Cx and CrAg	*C. neoformans*	L-AmB + FC -> FLZ	Alive	D49
Thyagarajan et al. [26] (USA)	75	M	DM, HTN, obesity	Severe	Yes	Disseminated	Blood Cx	*C. neoformans*	Nil	Dead	Postmortem
Traver et al. [30] (USA)	59	M	COPD, DM, CHF, liver cirrhosis, obesity	Severe	Yes	Pulmonary	BAL Cx	*C. neoformans*	L-AmB + FC -> FLZ	Dead	D10
Woldie et al. [34] (Canada)	24	M	Autoimmune hemolytic anemia	Severe	Yes	Disseminated	Blood Cx	*C. neoformans*	Nil	Dead	D23
Štingl et al. [35] (Czech)	60	M	HTN	Severe	Yes	Pulmonary	BAL Cx, CrAg	*C. neoformans*	L-Amb + FLZ	Dead	D22

BAL, bronchoalveolar lavage; CAD, coronary artery disease; CHF, congestive heart failure; CKD, chronic kidney disease; COPD, chronic obstructive pulmonary disease; CrAg, cryptococcus antigen; CSF, cerebrospinal fluid; Cx, culture; DM, diabetes mellitus; F, female; FC, flucytosine; FLZ, fluconazole; HTN, hypertension; ISZ, isavuconazole; L-AmB, liposomal amphotericin B; M, male; NA, not applicable; VCZ, voriconazole.

**Table 2:** Proposed treatment options for cryptococcal disease in HIV-negative COVID-19 patients [57,58].

● **CNS disease**
Induction therapy: - Amphotericin B (0.7–1 mg/kg/d) plus flucytosine (100 mg/kg/d) for at least 4 weeks - Amphotericin B (0.7–1 mg/kg/d) for 6–10 weeks for flucytosine-intolerant patients - Lipid formulation of amphotericin B (3–5 mg/kg/d) plus flucytosine (100 mg/kg/d) for at least 4 weeks for amphotericin B-intolerant patients - Lipid formulation of amphotericin B (3–5 mg/kg/d) for 6–10 weeks for flucytosine– and amphotericin B–intolerant patients Consolidation therapy: fluconazole (400 mg/d) for minimum 8 weeks Maintenance therapy: fluconazole (200 mg/d) for 6–12 months
● **Isolated pulmonary disease**
Mild-to-moderate symptoms or culture-positive specimen from this site: - Fluconazole, 200–400 mg/d for 6 months ^a^ - Itraconazole, 200–400 mg/d for 6 months ^a,b^ - Amphotericin B, 0.5–1 mg/kg/d (total, 1000–2000 mg) Severe symptoms and immunocompromised hosts: - Treat like CNS disease
● **Isolated cryptococcemia**
- Treat like CNS disease

^a^ Duration of therapy may be shortened to 3 months or extended to 12 months, which is based on resolution of disease. ^b^ Voriconazole (400 mg/d) and posaconazole (800 mg/d) are acceptable.

## Data Availability

All data were obtained from the original studies.
